# Perceptions on Academic Rhinologist Compensation Models: An ARS Survey

**DOI:** 10.1002/oto2.70107

**Published:** 2025-04-21

**Authors:** Kiran Abraham‐Aggarwal, Xiaoxuan Chen, Daniel J. Spertus, Shriya Suresh, Andrew B. Yang, Ashutosh Kacker

**Affiliations:** ^1^ School of Industrial and Labor Relations Cornell University Ithaca New York USA; ^2^ Department of Otolaryngology–Head and Neck Surgery Weill Cornell Medicine New York New York USA; ^3^ Department of Otolaryngology–Head and Neck Surgery, and MD Program Weill Cornell Medicine New York New York USA; ^4^ Department of Medicine Weill Cornell Medicine New York New York USA; ^5^ Department of Nutritional Sciences Cornell University Ithaca New York USA; ^6^ Harvard Medical School, Osher Center for Integrative Medicine Boston Massachusetts USA

**Keywords:** academics, compensation, fellows, otolaryngology, rhinologist

## Abstract

**Objective:**

To evaluate the perceptions of American Rhinologic Society (ARS) members on the compensation models of academic rhinologists and their impact on clinical practice, teaching, and academic responsibilities.

**Study Design:**

Survey study.

**Setting:**

Academic rhinologists across the United States who are members of the ARS.

**Methods:**

A twenty‐six‐question survey was distributed to 295 ARS members. The survey collected demographic information such as years of experience, geographic location, practice setting, and consultation volume. It also explored various compensation models and their impact on compensation, patient volume, case types, and the ability to support teaching and academic responsibilities.

**Results:**

Out of 295 surveyed ARS members, 107 responded (36%), and 80 academic rhinologists were included in the final sample. Respondents varied in experience and geographic distribution. Most respondents were salaried (69%), while 63% were under relative value units (RVU)‐based models, and 25% were under collections‐based models. Additionally, 66% reported poor or no support for research and educational activities. Compensation models were found to influence patient volume (28%), procedure choices (14%), and academic duties, with 55% of respondents indicating reduced engagement with students.

**Conclusion:**

Although a plurality of respondents (39%) believed that salaried models are most conducive to balancing academic and clinical responsibilities, survey findings highlight a dissonance. Respondents under collections‐based models were more likely to feel adequately supported (64.71%) compared to those under salaried or RVU‐based models. This suggests that although many perceive salaried models as ideal for balance, collections‐based models may better address financial and structural needs, emphasizing the importance of developing flexible, tailored compensation structures that align with individual and institutional goals while fostering academic productivity.

Otolaryngologists are the twelfth highest paid specialty in medicine, making $502,543/year on average.^1^ Within otolaryngology, rhinologists, who focus on nasal and sinus disorders, often achieve higher compensation levels, likely influenced by procedural reimbursement structures and work relative value units (wRVUs) associated with their cases.^2^ Academic rhinologists, who often hold teaching and research responsibilities in addition to their clinical duties, face compensation challenges similar to other academic physicians. As such, despite their significant contributions to medical education and advancing the field, their compensation often does not adequately reflect the additional time and effort required for teaching and academic activities.^3^, ^4^, ^5^ This disparity raises important questions about the value placed on educational roles within the medical profession and the need for more equitable compensation models.

In academic medicine, there are three financial models that are most common: salary‐based, wRVU‐based, or collections‐based.^6^ Compensation models are generally fixed at the institutional or departmental level, and individual rhinologists do not have the ability to choose a model.^7^ Today, most academic physicians are offered a salaried or salary plus bonus/incentive‐based model.^8^ This model can often be the most convenient and lucrative for new academic physicians as it offers a sense of pay security.^8^ Another model for rhinologists is wRVU‐based compensation; this model ties pay to the volume and complexity of services provided.^9^ The last option for most academic physicians is a collections‐based model. This is a combination of salary‐based and wRVU compensation and ties a physician's salary to their productivity.^10^ These salary calculations are often complicated and are tied to a physician's collections, where a percentage of their collections is earmarked for overhead expenses and the rest is allocated to a physician's compensation.^10^

To our knowledge, there are no studies that analyze academic rhinologist compensation models. To that end, we sought to understand academic rhinologists’ perceptions on their own salary models. We conducted a survey of all American Rhinologic Society (ARS) members in April and May 2024. This study examines how different compensation models influence academic rhinologists’ preferences, professional behaviors, and perceived support for clinical, teaching, and research responsibilities. Specifically, we hypothesize that compensation models with greater financial flexibility may be associated with higher perceived support for academic activities.

## Methods

This study aimed to understand the perceptions of academic rhinologists’ compensation models. In total, 295 individuals, from the ARS member database, were emailed including administrative, regular, fellow, international, emeritus, and retired members. The focus of this study was compensation; thus, subjects were not asked directly about their workload, level of burnout, or if they worked in academia. Additionally, it is important to note that this study did not differentiate between clinician‐scientists and clinician‐teachers; it concentrated more broadly on general academic clinicians. This study did not require Weill Cornell Medical College Institutional Review Board or ethics committee approval.

The questionnaire (Supplemental Figure S1, available online), consisting of 26 questions, gathered demographic data from surgeons, including years of experience, geographic region, practice setting, and consultation volume. Additionally, the study collected information designed to evaluate the various compensation models employed by academic rhinologists and the distribution or weighting of these models on their overall compensation. The study also aimed to explore how these compensation models might influence a rhinologist's patient volume and the types of cases they handle. Furthermore, the survey examined the impact of compensation models on a rhinologist's capacity to support their teaching and academic responsibilities.

To analyze the relationship between compensation model types and perceptions of support for academic and clinical responsibilities, we conducted three sets of Pearson's chi‐square tests. The first test assessed the association between the compensation model type (question 12) and respondents’ perceptions of how well their compensation model supports teaching and academic responsibilities (question 23). The second test evaluated the relationship between compensation model type (question 12) and how respondents allocate their time between teaching/academic duties and clinical practice (question 22). The third test examined the association between compensation model type (question 12) and perceptions of how well the model facilitates financial support for research and educational activities (question 29).

For individuals who selected multiple compensation models, each unique observation was treated as a separate data point to ensure all perspectives were accounted for in the analysis. As a result, the total number of observations for these analyses exceeds the number of survey respondents. These cross‐tabulations aimed to identify whether individuals under collections‐based compensation models felt more supported in teaching, research, or other academic activities compared to other compensation models.

## Results

### Respondent Demographics

A total of 107 members opened the survey, indicating a 36% response rate. From the 107 responses, 80 members were rhinologists who were employed or contracted with an academic hospital (Table 1). These 80 members were included in our final analyses. Years of experience ranging from less than 5 years to more than 20 years was fairly evenly distributed. Regarding academic titles, the largest portion (37%) of respondents held professor titles. Geographically, the respondents are spread across the United States, with the highest concentrations in the South (33%), Northeast (24%), and Midwest (22%). The majority (83%) practice in urban areas, with smaller numbers in suburban (14%) and rural (2.6%) regions.

**Table 1:** Survey Respondent Demographics^a^

Characteristics	N (%)
Years of experience	
Less than 5 y	17 (22%)
5‐10 y	17 (22%)
11‐20 y	21 (27%)
More than 20 years	**24 (30%)**
Title at academic hospital	
Instructor	1 (1.3%)
Assistant professor	27 (34%)
Associate professor	20 (25%)
Professor	**29 (37%)**
Professor Emeritus	1 (1.3%)
Research	1 (1.3%)
Other	3 (4%)
Geographic United States	
West	12 (16%)
Midwest	17 (22%)
Northeast	18 (24%)
South	**25 (33%)**
Pacific	1 (1.3%)
Puerto Rico	1 (1.3%)
Do not reside in the United States	2 (2.6%)
Practice region	
Rural	2 (2.6%)
Suburban	11 (14%)
Urban	**63 (83%)**

Bolded values indicate the response category with the greatest number of respondents.

^a^ Response totals may not add up to 80 due to nonresponses. Percentages may not add up to 100% either due to rounding, multiple selections, or nonresponses.

### Compensation Model Responses

Respondents could choose a combination of compensation models on a sliding 0% to 100% scale. As a result, some respondents chose more than one compensation model. A majority of respondents (69%) reported being salaried, 63% were under an relative value unit (RVU)‐based model, and 25% were under a collections‐based model (Table 2). Most rhinologists (75%) within the same practice have the same compensation model, and 43% mentioned their compensation model had changed over time. The impact of the compensation model on patient volume was noted by 28%, and 14% reported it affected their choice of procedures, though 86% stated their procedure types were not influenced by the model.

**Table 2:** Survey Responses Regarding Compensation Model^a^

Characteristics	N (%)
Compensation model	N = 67
Salaried	**46 (69%)**
RVU‐based	42 (63%)
Collections‐based	17 (25%)
Other	13 (19%)
Do all rhinologists at your practice have the same compensation model and weighting?	
Yes	**50 (75%)**
No	10 (15%)
Unknown	7 (10%)
Has your compensation model changed over time?	
Yes	29 (43%)
No	**38 (57%)**
Has the compensation model that you receive impacted your patient volume?	
Yes	18 (28%)
No	**47 (72%)**
Has the compensation model you receive affected the types of procedures you decide to take on?	
Yes	9 (14%)
No	**56 (86%)**
Which types of procedures are you most likely to perform under your current compensation model?	
Mostly common, or less complex procedures, or low RVU procedures	1 (2.6%)
A balanced mix of common and complex procedures	13 (20%)
Mostly complex or high RVU procedures	4 (6.2%)
Types of procedures are not influenced by my compensation model	**47 (72%)**
To what extent does the compensation model influence your decision‐making regarding patient care?	
Greatly influences to prioritize higher RVU procedures	2 (3.1%)
Somewhat influenced with balanced approach	14 (22%)
Does not influence my decision‐making	**48 (74%)**
Influences towards more conservative, nonsurgical management	1 (1.5%)
Under your compensation model, how do you allocate your time between teaching/academic duties and clinical practice?	
Predominantly teaching/academic duties	3 (4.7%)
Balanced between teaching/academic duties and clinical practice	**31 (48%)**
Predominantly clinical practice	30 (47%)
Entirely clinical practice	0 (0%)
To what extent does your current compensation model support your teaching and academic responsibilities?	
Fully supports	10 (16%)
Partially supports	**26 (41%)**
Does not support	23 (36%)
Actively hinders	5 (7.8%)
How does your compensation model impact your ability to engage with students and trainees?	
Enhanced engagement	7 (11%)
No impact on engagement	22 (34%)
Reduces engagement due to time constraints	**35 (55%)**
Severely limits engagement	0 (0%)
Does your institution's compensation model provide incentives for academic achievements (ie, research publications, securing grants)?	
Yes, substantial incentives	7 (11%)
Yes, minimal incentives	25 (39%)
No incentives	**32 (50%)**
How does your compensation model affect your flexibility to pursue academic endeavors outside of clinical duties?	
Greatly enhances flexibility	4 (6.3%)
Somewhat enhances flexibility	13 (20%)
No impact	21 (33%)
Limits flexibility	**26 (41%)**
Under your current compensation model, how would you rate your academic productivity?	
Significantly increased	3 (4.7%)
Slightly increased	10 (16%)
Unchanged	**30 (47%)**
Slightly decreased	16 (25%)
Significantly decreased	5 (7.8%)
Considering your balance between teaching/academic duties and clinical practice, which compensation model do you believe is most conducive to supporting academic rhinologists?	
Salaried model	**25 (39%)**
RVU‐based model	11 (17%)
Collections‐based model	11 (17%)
Other	17 (27%)
How well does your compensation model facilitate financial support for your research and educational activities?	
Very well to ample support	7 (11%)
Adequately to enough to meet basic needs	15 (23%)
Poorly to not enough support	**23 (36%)**
Not at all to no financial support	19 (30%)

Bolded values indicate the response category with the greatest number of respondents.

Abbreviation: RVU, relative value unit.

^a^ Response totals may not add up to 80 due to nonresponses. Percentages may not add up to 100% either due to rounding, multiple selections, or nonresponses.

**Figure 1:**
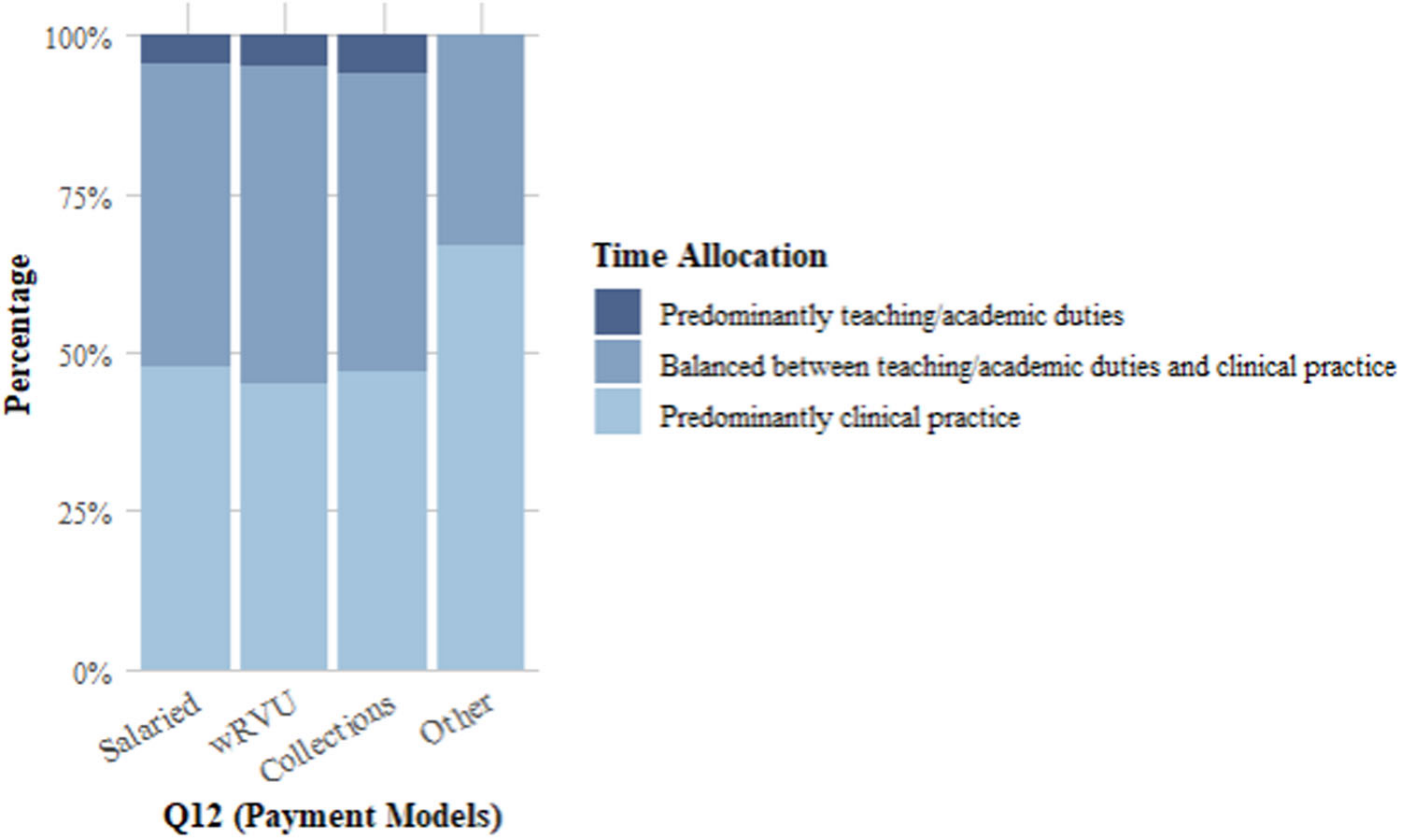
Variation in time allocation by compensation type. wRVU, work relative value unit.

When it came to decision‐making regarding patient care, the majority (74%) of respondents claimed no influence, 3.1% were greatly influenced to prioritize higher RVU procedures, whereas 22% had a balanced approach. Regarding time allocation, 48% balanced teaching/academic duties and clinical practice, with 47% focusing predominantly on clinical practice (Figure 1). Only 16% felt their model fully supported teaching and academic duties, whereas 36% felt unsupported, and 55% said their model reduced engagement with students and trainees.

In terms of incentives, 50% reported no incentives for academic achievements, and 41% said their compensation model limited flexibility for academic endeavors. Academic productivity under the current model for respondents remained unchanged for 47%, but 25% reported a slight decrease in productivity. A plurality (39%) believed that the salaried model best supports academic rhinologists, whereas financial support for research and educational activities was rated poorly by 36%, with 30% receiving no support at all.

### Chi‐Square Analyses

Three chi‐square tests were conducted to analyze the relationship between compensation model type and various professional responsibilities. Multiple observations were considered for respondents who selected more than one compensation model, resulting in a total number of observations exceeding the number of survey respondents. This approach allowed for the analysis of compensation models independently but may limit the ability to attribute findings specifically to individual models.

**Figure 2:**
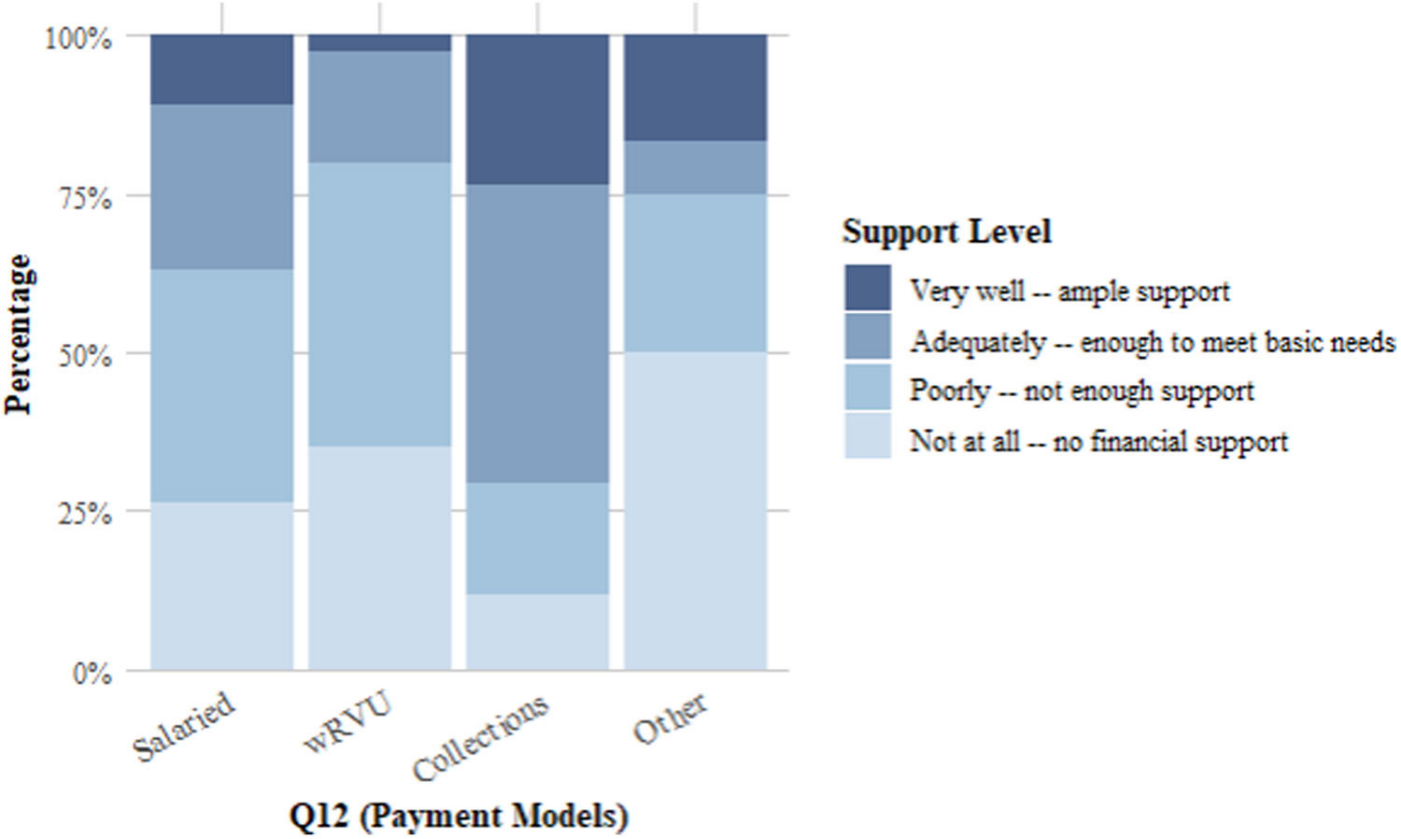
Do specific payment models more greatly influence support for teaching and academic duties? wRVU, work relative value unit.

The association between compensation model type (Q12) and perceptions of financial support for research and educational activities (Q29) was statistically significant (*χ*² = 18.39, df = 9, *P* = .03091) (Figure 2). Respondents under collections‐based models appear more likely to report higher levels of support, with 64.71% indicating their model provided “very well” or “adequately” for financial support. In contrast, respondents under RVU‐based or salaried models were more likely to report “poor” or “no” financial support, highlighting potential disparities in how different compensation models are perceived to support academic responsibilities.

The relationship between compensation model type (Q12) and time allocation between academic and clinical duties (Q22) did not yield statistically significant results (*χ*² = 2.1381, df = 6, *P* = .9066) (Table 2). Across all compensation models, most respondents reported a balance between teaching/academic responsibilities and clinical practice. No significant differences were observed in how compensation models influenced time allocation, suggesting that time distribution may not be heavily impacted by the type of compensation model.

The relationship between compensation model type (Q12) and its influence on decision‐making regarding patient care (Q21) also did not yield statistically significant results (*χ*² = 12.114, df = 9, *P* = .2069) (Supplemental Figure S2, available online). Respondents under collections‐based models uniformly reported that their compensation model did not influence decision‐making. In contrast, responses under RVU‐based models demonstrated greater variability, with some respondents indicating their model influenced them to prioritize higher RVU procedures or adopt a balanced approach.

### Cross‐Tabulations

**Table 3:** Cross‐Tabulations of Collections‐Based Model Respondents for Question 23

Q12	Q23	N	Percentage
3 (collections‐based)	1 (fully supports)	3	17.64706
3 (collections‐based)	2 (partially supports)	8	47.05882
3 (collections‐based)	3 (does not support)	6	35.29412

Table 3 further examines the relationship between compensation models and perceptions of financial support for academic and teaching activities (Q23). Respondents under collections‐based models were more likely to report “fully” or “partially” supported, with 64.71% selecting these categories. In comparison, respondents under salaried or RVU‐based models were more likely to indicate “does not” or “poorly” supports their teaching and academic responsibilities (Figure 2).

## Discussion

This study explores the impact of compensation models on professional activities and the perspectives of academic rhinologists on which models may support their roles. For academic rhinologists, surgeon compensation should ideally align the goals of surgeons with those of their institutions, encouraging high‐quality patient care, education, and innovation.^11^ To our knowledge, rhinologist perceptions of the included compensation models have not been investigated. Through this study, it has been observed that compensation models in academic medicine may play a role in shaping the career trajectories, job satisfaction, and productivity of physicians.

The findings of this study reveal important trends regarding how compensation models shape academic rhinologists’ perceptions of their professional support and responsibilities. Notably, the statistically significant association between compensation model type and perceptions of financial support for research and educational activities suggests that collections‐based models may provide greater alignment with the financial needs of academic responsibilities. Respondents under collections‐based models were significantly more likely to report higher levels of support, which may be attributed to the flexibility and resource allocation inherent to these models. This observation aligns with broader trends in academic medicine, where financial sustainability for research and education is increasingly tied to institutional and departmental incentives.^12^

An academic rhinologist's role is multifaceted, involving a balance between clinical practice and academic responsibilities. In our study, only 16% of respondents believed that their compensation model fully supported teaching and academic duties, whereas 36% felt it provided no support. These findings indicate that academic rhinologists are expected to fulfill teaching roles, yet for the survey respondents, current compensation models often do not provide adequate support for these activities. Survey results show that 55% of respondents reported a decrease in their engagement with students and trainees, which corresponds with their compensation model.

Despite the significant findings regarding financial support, no statistically significant differences were observed in the relationship between compensation model type and time allocation for academic versus clinical duties. This lack of differentiation may suggest that academic rhinologists, irrespective of their compensation model, experience similar expectations in balancing these responsibilities.

However, the observed variability in responses under RVU‐based models raises questions about how such models may implicitly influence the allocation of effort. Clemens et al and Reid et al highlight that financial incentives often have a measurable impact on behavior, particularly in systems where volume‐based or RVU‐linked incentives dominate, demonstrating the adage “money talks” in influencing clinical decision‐making.^13^, ^14^ Similarly, Maganty et al suggest that even modest financial incentives, such as those under the Merit‐Based Incentive Payment System, may subtly steer priorities despite appearing balanced.^15^ Although most respondents in this study reported a balanced approach, it is plausible that specific institutional contexts or individual contract arrangements amplify these effects, potentially leading to differing experiences with RVU‐based compensation models. Conversely, those operating under salary‐based or collection‐based models may be more accustomed to addressing concerns about compensation models influencing their decision‐making. As a result, they might be more inclined to report that they allocate time evenly or do not prioritize tasks based on reimbursement. This discrepancy underscores the need for further investigation into how variations within compensation models contribute to the perceived or actual balance between academic and clinical work.

In a study by Finn et al, interviews revealed that surgeons with clinical productivity‐based incentives reported a preference for increased volume of operative management, whereas surgeons with salaried models described their operative decision‐making as “pure clinical judgment.”^16^ In our study, 14% of respondents reported that their compensation model impacted the types of procedures they performed. This suggests that for a subset of rhinologists, compensation models might incentivize more complex or high RVU procedures. Moreover, only 2.6% were inclined to perform mostly common or low RVU procedures, whereas 20% maintained a balanced mix, underscoring that compensation models can subtly influence clinical decision‐making and patient care strategies.

Given these challenges, a plurality of respondents (39%) believed the salaried model is most conducive to supporting academic rhinologists. This preference suggests that a stable income, not directly tied to clinical productivity, allows for greater emphasis on teaching, research, and academic responsibilities. However, findings from this study highlight notable advantages of collections‐based models, particularly in providing financial support for research and educational activities. Respondents under collections‐based models were significantly more likely to report feeling “very well” or “adequately” supported (64.71%) compared to those under RVU‐based or salaried models. This indicates that, though some academic rhinologists feel as though a salaried model may be more conducive to balance, there is a dissonance that persists. In fact, a collections‐based model may better align with the financial needs of academic rhinologists, potentially offering a more structured framework for supporting their multifaceted roles.

Additionally, although surgeons with clinical productivity‐based incentives may increase clinical activities related to generating more revenue, these models may also be associated with higher rates of burnout, internal competition, and operative management.^17^ The variability observed in responses under RVU‐based models regarding decision‐making suggests that these systems may incentivize prioritization of higher RVU procedures, which could subtly influence clinical practice. In contrast, respondents under collections‐based models uniformly reported that their compensation model did not influence their decision‐making, possibly reflecting a better alignment of financial structures with clinical judgment.

These findings highlight the complexity of designing compensation models that balance institutional goals with the diverse responsibilities of academic rhinologists. Although collections‐based models appear to provide stronger support for research and educational activities, the salaried model's perceived stability and flexibility remain valued by a significant proportion of respondents. Future efforts should consider hybrid models that integrate the strengths of different compensation systems to address the unique demands of academic rhinology.

Our study has several limitations that impact the interpretation of the findings. Due to the nature of a survey, the presence of voluntary response bias can lead to skewed results, and social desirability bias may have caused respondents to underreport the influence of compensation on clinical decision‐making. Furthermore, the survey focused primarily on perceptions and opinions, without incorporating objective data on procedural RVUs, bonus structures, or compensation targets, which could provide a more comprehensive understanding of the relationship between compensation and clinical behavior. For example, prior research, such as Poteet et al, demonstrates that productivity‐based compensation models are associated with increased RVU generation, a finding that may contrast with the self‐reported perceptions in this study.^18^ This highlights the need for future work that combines quantitative data with survey results.

Additionally, our survey did not differentiate between academic and clinical tracks, nor did it address the diversity of academic duties, which can significantly influence workload and compensation. Variations in expectations for FTE contributions, as well as differences in research focus, teaching responsibilities, and clinical workload, were not accounted for, limiting the generalizability of the findings. The survey also did not include questions on gender, which may play a role in compensation disparities, or on additional factors such as burnout, stress, or workload, which are critical to understanding job satisfaction and productivity. Finally, the absence of granular data on compensation components, such as RVU targets or bonuses beyond base salary, limits the study's ability to fully assess the nuanced relationship between compensation models and professional activities.

Future studies should aim to address the limitations of our research by incorporating both subjective perceptions and objective metrics to provide a more holistic understanding of how compensation models influence academic rhinologists. For example, integrating procedural and financial data, such as RVU generation, bonus structures, and compensation targets, would allow for a clearer assessment of the relationship between compensation models and professional behaviors. Studies could also explore how specific compensation structures impact the allocation of time between clinical practice, teaching, and research. This information would be critical in evaluating whether certain models incentivize or hinder engagement with academic responsibilities, including medical education and scholarly productivity.

Additionally, future research should account for the diversity in academic and clinical tracks, as these roles involve different expectations for FTE contributions, workload, and productivity benchmarks. A detailed analysis of compensation models across subspecialties within otolaryngology would help clarify the extent to which procedural reimbursement disparities, such as those highlighted by differences in RVU assignments for rhinologic versus otologic procedures, drive inequities in compensation. This could inform policy recommendations for standardizing compensation frameworks to better reflect the complexity and value of various clinical and academic roles.

Gender, as well as other sociodemographic factors, should also be incorporated into future surveys and analyses, as they may contribute to disparities in compensation and career advancement. Exploring how compensation models interact with these factors could provide insights into addressing inequities and fostering inclusivity within academic medicine.

Lastly, longitudinal studies could investigate the long‐term effects of different compensation models on job satisfaction, career trajectories, and burnout among academic rhinologists. Tracking these outcomes over time would provide valuable data on the sustainability of current compensation practices and the effectiveness of proposed reforms. For example, studies could evaluate whether transitioning from productivity‐based to salaried or hybrid models leads to improved engagement in academic activities, enhanced career satisfaction, and reduced burnout.

## Conclusion

Compensation models significantly impact academic rhinologists by influencing their clinical, teaching, and research activities. Although a plurality of respondents (39%) believed that salaried models are most conducive to balancing academic and clinical responsibilities, survey findings highlight a dissonance. Respondents under collections‐based models were more likely to feel adequately supported (64.71%) compared to those under salaried or RVU‐based models. This suggests that although many perceive salaried models as ideal for balance, collections‐based models may better address financial and structural needs, emphasizing the importance of developing flexible, tailored compensation structures that align with individual and institutional goals while fostering academic productivity. Developing compensation models that adequately support teaching and research activities, while maintaining high‐quality patient care, is essential for fostering a thriving academic environment.

## Level of Evidence

Based on the Oxford Centre for Evidence‐Based Medicine's 2011 Level of Evidence Guide, our study is of level 3 evidence.

## Author Contributions

**Kiran Abraham‐Aggarwal**, conceptualization, methodology, software, validation, formal analysis, investigation, resources, data curation, original draft, review and editing, visualization, project administration; **Xiaoxuan Chen**, methodology, validation, formal analysis, investigation, data curation, original draft, review and editing, visualization; **Daniel J. Spertus**, methodology, investigation, formal analysis, review and editing, visualization; **Shriya Suresh**, investigation, review and editing; **Andrew B. Yang**, review and editing, visualization; **Ashutosh Kacker**, validation, original draft, review and editing, project administration.

## Disclosures

### Competing interests

None.

### Funding source

NA.

## Supporting information

Supplemental File 1: Survey Sent to ARS Members.

Supplemental File 2: Extent to Which Compensation Model Influences Decision‐Making Within Each Compensation Model.

## References

[1] Condon A . 40 physician specialties ranked by compensation. Becker's Hospital Review. Accessed May 25, 2024. https://www.beckershospitalreview.com/compensation-issues/physician-pay-up-5-9-but-inflation-eats-into-real-income-doximity.html

[2] Kondamuri NS , Miller AL , Rathi VK . Compensation rates for otolaryngologic procedures under the Medicare physician fee schedule in 2018. Laryngoscope. 2021;131(6):E1785‐E1791. 33331651 10.1002/lary.29328

[3] Rabinowitz E . The Tradeoffs Between Academic and Private Practice. Medical Economics; 2024. https://www.medicaleconomics.com/view/the-tradeoffs-between-academic-and-private-practice

[4] Brown JB , Fluit M , Lent B , Herbert C . Seeking balance: the complexity of choice‐making among academic surgeons. Acad Med. 2011;86(10):1288‐1292. 10.1097/ACM.0b013e31822c124a 21869660

[5] Hull BP , Darrow DH , Derkay CS . The financial value of fellowship training in otolaryngology. Otolaryngol Head Neck Surg. 2013;148(6):906‐911. 10.1177/0194599813482094 23554112

[6] Emery SE . Physician incentives for academic productivity: an analysis of orthopaedic department compensation strategies. J Bone Joint Surg. 2006;88(9):2049‐2056. 10.2106/jbjs.E.00243 16951123

[7] Bucci RV , Bucci RV . Physician employment and compensation. In: Medicine and Business: A Practitioner's Guide, 2014:149‐159.

[8] Sturm MR . Physician compensation: designing the “best‐fit” plan. Oncol Issues. 2012;27(2):28‐35.

[9] Slater BJ , Collings AT , Corvin C , Kandel JJ . Value‐based surgery physician compensation model: review of the literature. J Pediatr Surg. 2022;57(9):118‐123. 10.1016/j.jpedsurg.2022.01.009 35093253

[10] Darves B . Physician compensation models: the basics, the pros, and the cons. New Engl J Med. 2004. http://www.nejmjobs.org/resource_center/Physician_Compensation.asp

[11] Reiter AJ , Warner SG , Chen H , et al. Translating the value of the academic surgeon into salary, time, and resources. J Surg Res. 2023;285:A1‐A6. 36682973 10.1016/j.jss.2022.12.039

[12] Ensuring the financial sustainability of academic medical centers. McKinsey & Company. 2025. https://www.mckinsey.com/industries/healthcare/our-insights/ensuring-the-financial-sustainability-of-academic-medical-centers

[13] Clemens J , Gottlieb JD . Do physicians' financial incentives affect medical treatment and patient health? Am Econ Rev. 2014;104(4):1320‐1349. 25170174 10.1257/aer.104.4.1320 PMC4144420

[14] Reid RO , Tom AK , Ross RM , Duffy EL , Damberg CL . Physician compensation arrangements and financial performance incentives in US health systems. JAMA Health Forum. 2022;3(1):e214634. 10.1001/jamahealthforum.2021.4634 35977236 PMC8903115

[15] Maganty A , Shah AA , Hill D , Golla V . Financial implications of the merit‐based incentive payment system for surgical health care professionals. JAMA Surg. 2024;159(2):221‐223. 10.1001/jamasurg.2023.5638 37991752 PMC10867682

[16] Finn CB , Syvyk S , Bergmark RW , et al. Perceived implications of compensation structure for academic surgical practice: a qualitative study. JAMA Surg. 2024;159(1):106‐107. 37878286 10.1001/jamasurg.2023.4669 PMC10600719

[17] Shanafelt TD , Balch CM , Bechamps GJ , et al. Burnout and career satisfaction among American surgeons. Ann Surg. 2009;250(3):463‐471. 19730177 10.1097/SLA.0b013e3181ac4dfd

[18] Poteet SJ , Harzman A , Chao AH . Surgical residents' perceptions of the impact of productivity‐based faculty compensation at an academic medical center. J Surg Res. 2021;259:114‐120. 10.1016/j.jss.2020.11.025 33279836

