# Supplementary material for: Perceptions on Academic Rhinologist Compensation Models: An ARS Survey
Source: OTO Open. 2025 Apr 21;9(2):e70107. doi: 10.1002/oto2.70107 (PMC12010749; doi:10.1002/oto2.70107)
Supplement: Supplementary file 1 — Supplemental File 1: Survey Sent to ARS Members. [file OTO2-9-e70107-s001.docx]

Academic Rhinologist Compensation Model Survey

Start of Block: Default Question Block

Q1 This study examines the structure of physician compensation in the field of academic medicine, specifically within otolaryngology and its subspecialty, rhinology. Rhinology stands out for generating higher Relative Value Units (RVUs) compared to other ENT areas, indicating the high value of its procedures. In academic settings, faculty members often engage in teaching, a task that consumes significant time and resources but does not directly contribute to their income. This survey focuses on three main compensation models: salaried, where physicians receive a fixed income; RVU-based, which ties pay to the volume and complexity of services provided; and collection-based, where compensation depends on the revenue generated from patient services. Our goal is to evaluate these models to determine the most fitting approach for rewarding academic physicians, taking into account their unique balance of clinical and educational duties.

Q2 I understand participation is voluntary and that my responses will remain confidential, and data will be de-identified. I understand that by completing the survey, I agree to participate in this study.

- Yes (1)
- No (2)

Skip To: End of Survey If Q2 = No

| Page Break |  |
| --- | --- |

Q3 Are you employed or do you contract with an academic hospital?

- Yes (1)
- No (2)

Skip To: End of Survey If Q3 = No

Q4 Are you a rhinologist?

- Yes (1)
- No (2)

Skip To: End of Survey If Q4 = No

| Page Break |  |
| --- | --- |

Q5 How many years of experience do you have?

- Less than 5 (1)
- 5-10 years (2)
- 11-20 years (3)
- More than 20 years (4)

Q6 What is your title at your academic hospital? (select all that apply)

- Instructor (1)
- Assistant Professor (2)
- Associate Professor (3)
- Professor (4)
- Professor Emeritus (5)
- Research (6)
- Other (please specify) (7) __________________________________________________

End of Block: Default Question Block

Start of Block: State

Q1 What is the geographic location of your practice?

▼ Alabama (1) ... I do not reside in the United States (53)

Q9 Do you practice in a rural or urban setting?

- Rural (1)
- Suburban (2)
- Urban (3)

Q10 How many patients do you consult with per week?

- Less than 10 (1)
- 10-20 (2)
- 21-50 (3)
- More than 50 (4)

| Page Break |  |
| --- | --- |

Q11 The next few questions will ask about the compensation model that you receive. Again, your responses will remain confidential and data will be de-identified. You have agreed that your participation is voluntary.

| Page Break |  |
| --- | --- |

Q12 What model is your current compensation based on? (select all that apply)

- Salaried (1)
- RVU based (2)
- Collections based (3)
- Other (please specify) (4) __________________________________________________

Q13 If you selected multiple models, please describe the distribution or weighting among them.

|  | 0 | 10 | 20 | 30 | 40 | 50 | 60 | 70 | 80 | 90 | 100 |
| --- | --- | --- | --- | --- | --- | --- | --- | --- | --- | --- | --- |

| Salaried () | 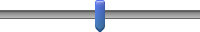 |
| --- | --- |
| RVU based () | 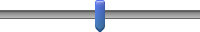 |
| Collections based () | 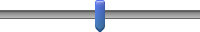 |

Q14 Do all rhinologists at your practice have the same compensation model and weighting?

- Yes (1)
- No (2)
- Unsure (3)

Display This Question:

If Q14 = No

Q15 How did you choose which compensation model to receive?

________________________________________________________________

Q16 Has your compensation model changed over time?

- Yes (1)
- No (2)

Display This Question:

If Q16 = Yes

Q17 Please elaborate on how your compensation model has changed over time.

________________________________________________________________

| Page Break |  |
| --- | --- |

Q18 Has the compensation model that you receive impacted your patient volume?

- Yes (1)
- No (2)

Q19 Has the compensation model you receive affected the types of procedures you decide to take on?

- Yes (1)
- No (2)

Q20 Which types of procedures are you most likely to perform under your current compensation model?

- Mostly common, or less complex procedures, or low RVU procedures (1)
- A balanced mix of common and complex procedures (2)
- Mostly complex or high RVU procedures (3)
- The type of procedures are not influenced by my compensation model (4)

Q21 To what extent does the compensation model influence your decision-making regarding patient care?

- Greatly influences to prioritize higher RVU procedures (1)
- Somewhat influenced with a balanced approach (2)
- Does not influence my decision making (3)
- Influences towards more conservative, non-surgical management (4)

| Page Break |  |
| --- | --- |

Q22 Under your compensation model, how do you allocate your time between teaching/academic duties and clinical practice?

- Predominantly teaching/academic duties (1)
- Balanced between teaching/academic duties and clinical practice (2)
- Predominantly clinical practice (3)
- Entirely clinical practice (4)

Q23 To what extent does your current compensation model support your teaching and academic responsibilities?

- Fully supports (1)
- Partially supports (2)
- Does not support (3)
- Actively hinders (4)

Q24 How does your compensation model impact your ability to engage with students and trainees?

- Enhances engagement (1)
- No impact on engagement (2)
- Reduces engagement due to time constraints (3)
- Severely limits engagement (4)

Q25 Does your institution's compensation model provide incentives for academic achievements (i.e., research publications, securing grants)?

- Yes, substantial incentives (1)
- Yes, minimal incentives (2)
- No incentives (3)
- Not applicable to my role (4)

Q26 How does your compensation model affect your flexibility to pursue academic endeavors outside of clinical duties?

- Greatly enhances flexibility (1)
- Somewhat enhances flexibility (2)
- No impact (3)
- Limits flexibility (4)

Q27 Under your current compensation model, how would you rate your academic productivity?

- Significantly increased (1)
- Slightly increased (2)
- Unchanged (3)
- Slightly decreased (4)
- Significantly decreased (5)

Q28 Considering your balance between teaching/academic duties and clinical practice, which compensation model do you believe is most conducive to supporting academic rhinologists?

- Salaried model (1)
- RVU-based model (2)
- Collections-based model (3)
- Other (please specify) (4) __________________________________________________

Q29 How well does your compensation model facilitate financial support for your research and educational activities?

- Very well—ample support (1)
- Adequately—enough to meet basic needs (2)
- Poorly—not enough support (3)
- Not at all—no financial support (4)

End of Block: State
