# Supplementary material for: Perceptions on Academic Rhinologist Compensation Models: An ARS Survey
Source: OTO Open. 2025 Apr 21;9(2):e70107. doi: 10.1002/oto2.70107 (PMC12010749; doi:10.1002/oto2.70107)

Supplemental File 2: Extent to Which Compensation Model Influences Decision-Making Within Each Compensation Model


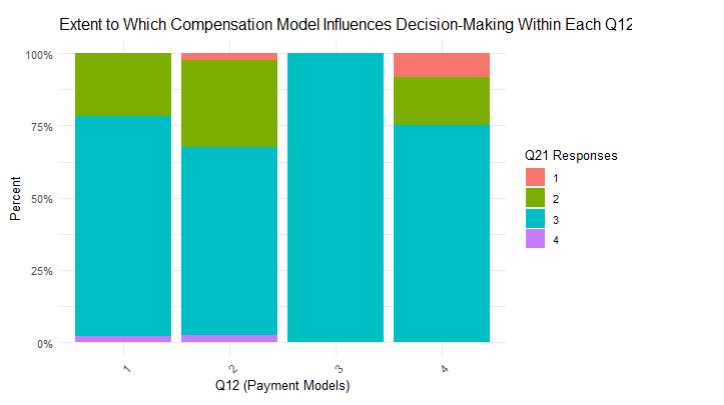

Supplement: Supplementary file 2 — Supplemental File 2: Extent to Which Compensation Model Influences Decision‐Making Within Each Compensation Model. [file OTO2-9-e70107-s002.docx]
